# Comparing Transcriptomes Reveals Key Metabolic Mechanisms in Superior Growth Performance Nile Tilapia (*Oreochromis niloticus*)

**DOI:** 10.3389/fgene.2022.879570

**Published:** 2022-07-12

**Authors:** Binglin Chen, Wei Xiao, Zhiying Zou, Jinglin Zhu, Dayu Li, Jie Yu, Hong Yang

**Affiliations:** Key Laboratory of Freshwater Fisheries and Germplasm Resources Utilization, Ministry of Agriculture and Rural Affairs, Freshwater Fisheries Research Center, Chinese Academy of Fishery Sciences, Wuxi, China

**Keywords:** tilapia, growth, energy metabolism, transcriptomes, substance metabolism

## Abstract

Metabolic capacity is intrinsic to growth performance. To investigate superior growth performance in Nile tilapia, three full-sib families were bred and compared at the biochemical and transcriptome levels to determine metabolic mechanisms involved in significant growth differences between individuals under the same culture environment and feeding regime. Biochemical analysis showed that individuals in the higher growth group had significantly higher total protein, total triglyceride, total cholesterol, and high- and low-density lipoproteins, but significantly lower glucose, as compared with individuals in the lower growth group. Comparative transcriptome analysis showed 536 differentially expressed genes (DEGs) were upregulated, and 622 DEGs were downregulated. These genes were significantly enriched in three key pathways: the tricarboxylic acid cycle (TCA cycle), fatty acid biosynthesis and metabolism, and cholesterol biosynthesis and metabolism. Conjoint analysis of these key pathways and the biochemical parameters suggests that Nile tilapia with superior growth performance have higher ability to consume energy substrates (e.g., glucose), as well as higher ability to biosynthesize fatty acids and cholesterol. Additionally, the fatty acids biosynthesized by the superior growth performance individuals were less active in the catabolic pathway overall, but were more active in the anabolic pathway, and might be used for triglyceride biosynthesis to store excess energy in the form of fat. Furthermore, the tilapia with superior growth performance had lower ability to convert cholesterol into bile acids, but higher ability to convert it into sterols. We discuss the molecular mechanisms of the three key metabolic pathways, map the pathways, and note key factors that may impact the growth of Nile tilapia. The results provide an important guide for the artificial selection and quality enhancement of superior growth performance in tilapia.

## Introduction

Nile tilapia (*Oreochromis niloticus*) is one of the most farmed fish worldwide, with 4.53 million tonnes produced in 2018 accounting for 8.3% of the world’s total aquaculture production (The State of World Fisheries and Aquaculture 2020, 2020). Although there are established breeding models and management methods for this species, and it has long been selectively bred for growth performance (Circa et al., 1995), large differences in growth still occur among individuals of the same genetic background and under the same rearing conditions (Chen et al., 2021), which effects their average breeding cycle and size, in turn making it difficult to control the costs of breeding and processing. Compared with higher-priced fish fillets, smaller tilapia was more often made into lower-priced fish meal, fish oil, or other byproducts, which has a great impact on the economic benefits of the tilapia industry. Simple phenotypes, such as body weight and activity, are the criteria still used by the majority of farmers to judge and establish quality breeding stocks, but, with the expansion of aquaculture and the increase in quality requirements, such criteria are no longer sufficient to meet farmers’ requirements for selecting superior breeding stock (Neira et al., 2004; Rutten et al., 2005). Therefore, a better understanding of the molecular mechanisms of tilapia growth will provide more possibilities to develop this industry.

The growth of organisms is inextricably linked to metabolism as the ordered series of chemical reactions continuously needed to sustain life and enable the body to grow, reproduce, and maintain stability of its internal environment in response to environmental change (Enquist et al., 2003). Cell metabolism can be divided into pathways of substance metabolism and energy metabolism, which denote the ability of cells to exchange substances and transfer energy, and ultimately gives the organism its different phenotypic traits (DeBerardinis et al., 2008). Most studies have described key metabolic mechanisms that are vital to animal growth, but, owing to the complex metabolic network of an organism, elaboration of the relationship between metabolism and growth has largely focused on descriptions of signaling pathways (Yang et al., 2008; Mihaylova and Shaw, 2011; Wang M.-C. et al., 2022, Wang et al., 2022 M.; Yin et al., 2022). However, with the advancement of genomics and transcriptome analysis now provide feasibility for the exploration of key genes that determine key mechanisms in biological metabolism.

The development of a range of high-throughput sequencing techniques that includes transcriptome sequencing (RNA-seq) provides possibilities for relating an increasing number of phenotypic traits and molecular function. With the completion and updating of the tilapia reference genome, comparative RNA-seq-based analyses have revealed a variety of molecular mechanisms of tilapia, including: disease resistance (Wang et al., 2016), sexual differentiation (Tao et al., 2018), body color (Wang et al., 2018), and environmental adaptations (Liu et al., 2018), but the metabolic mechanisms of Nile tilapia with superior growth performance and the possible relationships to growth have been rarely reported.

Here, we report RNA-seq data obtained from full-sib families of Nile tilapia that showed significant growth differences under the same culture environment and feeding regime. We aimed to screen for differentially expressed genes (DEGs) between tilapia with higher average body weight (faster growth) and lower average body weight (slower growth), to identify key metabolic genes that may be associated with body weight gain, and ultimately to describe and explain the metabolic mechanisms that involve a range of key genes. The results provide insight into the genetic and molecular mechanisms potentially associated with effective weight gain in Nile tilapia with superior growth performance.

## Materials and Methods

### Animal Breeding and Sampling

The study was conducted according to the guidelines of the Declaration of Helsinki, and approved by the ethics committee of laboratory animal welfare and ethics of Freshwater Fisheries Research Center, Chinese Academy of Fishery Sciences (FFRC, CAFS), No. SYXK (SU) 2017-0007. Nile tilapia were obtained from three full-sib families (F1, F2, F3) from the tilapia genetic breeding base of the Freshwater Fisheries Research Centre of the Chinese Academy of Fishery Sciences. The tilapia used in the study was the GIFT population, which consisted of 60 families at the time of introduction. FFRC mixed all the families and randomly retained 5,000 offspring every 3 years but did not select for growth performance, in order to ensure that genetic diversity was not destroyed. The three tilapia pairs used to establish the full-sib families in this study were selected from the 4th generation (born in 2017) GIFT population. In May 2019, three pairs of 2-year-old Nile tilapia (♀:♂ = 1:1) were artificially inseminated and incubated to establish full-sib families, and all fertilized eggs were broken within one week and standardized for 2 weeks for fry breeding. Next, 500 juveniles of body weight (BW) 2.0 ± 0.5 g were randomly selected from each family and then released into a 35 m^2^ pond for rearing; each family was raised in a separate pond with water temperature 28-32°C, about 13 h of natural daylight per day, pH of 7.0-8.0, and dissolved oxygen >5.0 mg/L. These juveniles were hand-fed a commercial feed of expanded (floating) pellets (30% crude protein, Nanjing ADM Animal Nutrition Co., Ltd., Nanjing, China), twice a day (at 8:00 and 18:00) until apparent satiation, for 3 months; satiation was determined by observing when feed remained after 60 min (uneaten food was then removed) according to Chen et al. (Chen et al., 2021).

**FIGURE 1:**
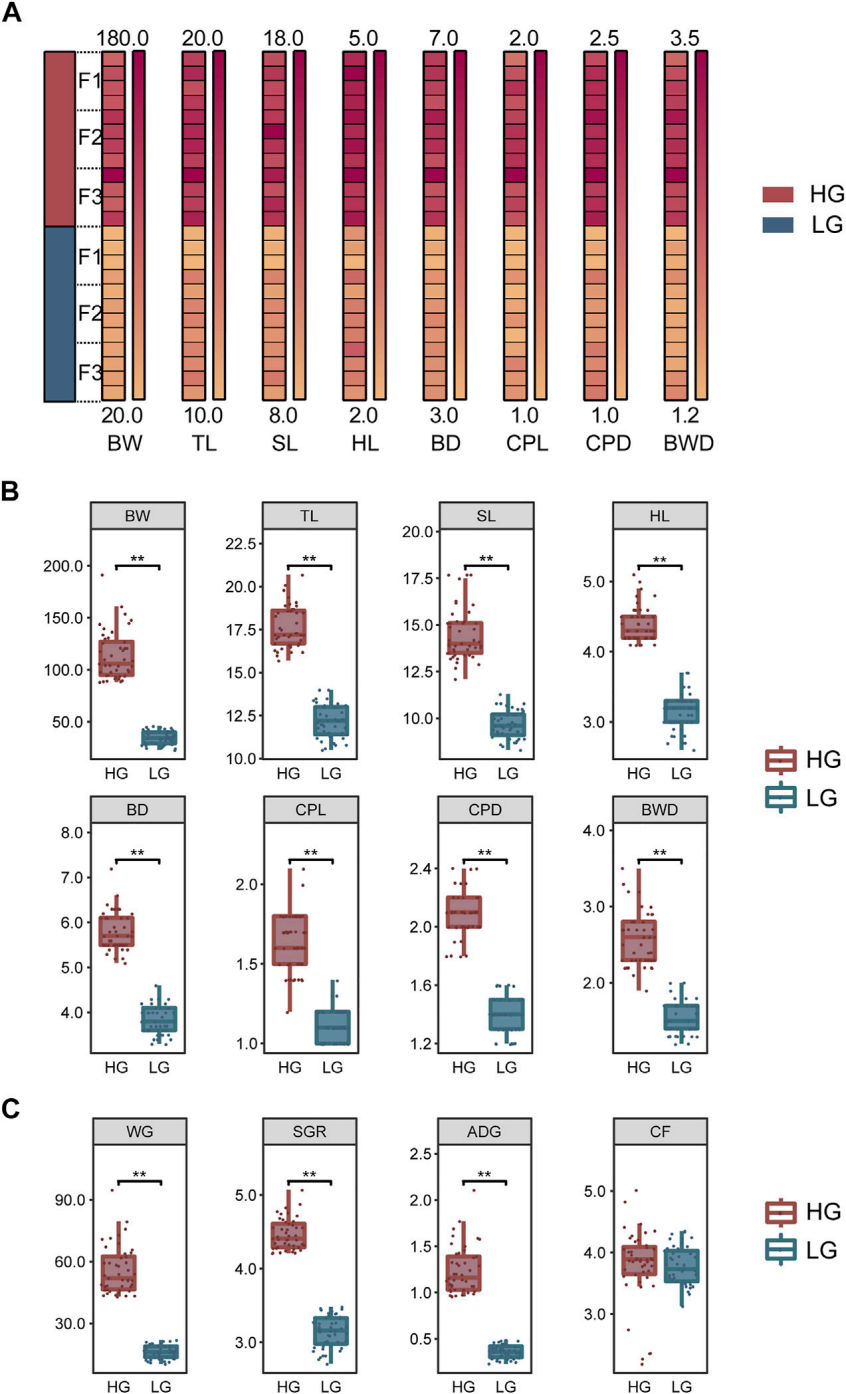
Growth traits and analysis of significant differences among samples. **(A)** Differences in growth traits of the Nile tilapia used for transcriptome sequencing. **(B)** Significant differences in growth traits between the higher growth (HG) and lower growth (LG) groups of tilapia (*n* = 45, each group). **(C)** Differences in weight gain (WG), specific growth rate (SGR), average daily growth (ADG), and condition factor (CF) between the HG and LG groups (*n* = 45, each group). Levene’s test was used to assess the equality of variances in the data, and the independent samples *t*-test showed a normal distribution (*p* > 0.05). **p < 0.01.

To exclude the influence of sexual dimorphism in tilapia, the sex of the individuals in each family was identified and their growth traits were measured before tissue and blood sampling. Fifteen males with the highest growth performance (i.e., maximum weight) and 15 males with the lowest growth performance (minimum weight) were then selected in each family. Thus, a total of 90 extreme-growth individuals were obtained from the three families: 45 individuals with high growth performance were classified as the higher growth group (HG), and 45 individuals with low growth as the lower growth group (LG). These groups showed normal distribution (*p* > 0.05) and significant differences in BW, total length (TL), standard length (SL), head length (HL), body depth (BD), caudal peduncle length (CPL), caudal peduncle depth (CPD), and body width (BWD) (*p* < 0.01) (Figure 1B). In addition, we add weight gain (WG) [(Final body weight− Initial body weight)/Initial body weight], specific growth rate (SGR) {100×[ln(Final body weight)− ln(Initial body weight)]/No. of days}, average daily growth (ADG) [(Final body weight− Initial body weight)/No. of days], and condition factor (CF) (100×BW/BL^3^) to provide a more comprehensive description of growth according to Du and Turchini (2022) (Figure 1C). All growth data were shown in Supplementary Table S1. Four individuals were randomly selected from the HG and LG in each family (i.e., a total of 12 HG individuals and 12 LG individuals from three families) for transcriptomic sequencing and subsequent biochemical parameters and qRT-PCR analysis; The mean BW (Mean ± SD) of the individuals used for sampling was 125.4 ± 25.2 g and 35.9 ± 7.6 g for HG and LG, respectively (Figure 1A). These 24 fish were anesthetized with Tricaine (MS-222) at a concentration of 13.5 g/m^3^ in 25°C water, to exclude the effect of stress on the experiment. Liver tissue was collected and stored at −80°C for RNA extraction and transcriptome analysis. Blood was collected (>500 µL) and centrifuged (12,000 g, 5 min, 4°C), and the supernatant was stored at −80°C for the biochemical analysis.

### Biochemical Assays

The 24 samples (12 from HG and 12 from LG) used for transcriptome analysis were continued to be used for blood biochemical analysis to ensure the accuracy and consistency of the experiment. The activities of total protein (TP), total triglyceride (TG), total cholesterol (TC), glucose (Glu), high-density lipoprotein (HDL), and low-density lipoprotein (LDL) were measured using commercial kits manufactured by Mindray Biomedical Electronics Co., Ltd. (Shenzhen, China), the volumes used for parameter tested was consistent between each sample. All kits were passed the quality control of the manufacturer. The coefficient of variation between replicates and the relative deviation between batches are less than 3 and 5%, respectively. And these kits have been shown to be widely used for the determination of fish biochemical parameters (Xiao et al., 2022).

### RNA Extraction, Library Preparation, and Sequencing

For transcriptome analysis, each group contained three samples (higher growth group: HG1, HG2, and HG3; lower growth group: LG1, LG2, and LG3), and each sample mixed liver tissue from four individuals showing the same growth performance and from the same family. Total RNA in liver tissue was isolated with TRIzol reagent (Invitrogen, Carlsbad, United States), following the manufacturer’s instructions, checked using 1% agarose gel electrophoresis, and its concentration and purity determined with a NanoDrop™ Lite spectrophotometer (Thermo Fisher Scientific, Waltham, United States). The RNA-seq transcriptome library was prepared using a TruSeq RNA Sample Prep Kit (Illumina, San Diego, United States) with 1 μg of total RNA. The libraries were sequenced on an Illumina HiSeq 4000 platform (Illumina, San Diego, United States).

### Identification and Functional Annotation of DEGs

Before data analysis, quality control of raw sequencing data using FASTP software (Chen et al., 2018). To obtain high-quality data, we removed adapter sequences, sequences of unknown nucleotides (>10%), and low-quality reads (Q-value ≤ 20). The clean reads were mapped to the reference genome (NCBI: GCF_001858045.2, *Oreochromis niloticus*) (Conte et al., 2017) using HISAT2.2.4 (Kim et al., 2015). To quantify the expression abundance and variation of each transcription region, we used RSEM software to calculate transcripts per million (TPM) (Li and Dewey, 2011). The differentially expressed genes (DEGs) between two groups were analyzed using DESeq2 software (Love et al., 2014). Gene expression with a false discovery rate (FDR) of <0.05 and fold change (FC) of >2 or <0.5 was considered to be a significant difference. To recognize the main biological functions of DEGs, we applied Gene Ontology (GO) enrichment analysis with GOseq software (Young et al., 2010); to identify the enrichment signaling pathways of DEGs, Kyoto Encyclopedia of Genes and Genomes (KEGG) pathway analysis was applied using KOBAS 2.0 software (Xie et al., 2011). FDR ≤0.05 was taken as the threshold for GO and KEGG enrichment. The RNA-Seq data has been submitted in Sequence Read Archive (SRA) database (No. PRJNA787719).

### Correlation Network Analysis

Growth performance, DEGs with research potential (29 DEGs that were significantly enriched in three major metabolic pathways: TCA cycle, Fatty acid biosynthesis and metabolism, and cholesterol biosynthesis and metabolism), and biochemical parameters were included in correlation analysis. Significant correlations (*p* < 0.01) with Pearson’s correlation coefficient more than 0.80 were used for network visualization in Gephi 0.9.2 (Lin et al., 2020).

### Quantitative Real-Time PCR Analysis

The 24 samples (12 from HG and 12 from LG) used for transcriptome analysis were continued to be used for qRT-PCR analysis to ensure the accuracy and consistency of the experiment. We performed qRT-PCR on DEGs that are significantly enriched in three major metabolic pathways: TCA cycle, Fatty acid biosynthesis and metabolism, and cholesterol biosynthesis and metabolism, to verify their relative expression trends. First-strand cDNA for each sample was synthesized with equal amounts of 900 ng of total RNA (The RNA extraction method used for gene expression is the same as described in 2.3) using a PrimeScript™ RT Reagent Kit (TaKaRa, Dalian, China); mRNA expression levels of DEGs in the liver tissue of the different groups were measured using quantitative real-time PCR (qRT-PCR) in a 7900HT Fast Real-Time PCR System (Applied Biosystems, Waltham, United States). The qRT-PCR was performed using SYBR^®^ Green qPCR Master Mix (Vazyme, Nanjing, China) in a 20 μL total reaction volume containing 10 μL of SYBR^®^ Green qPCR Master Mix, 1 μL of cDNA template, 0.4 μL of each primer (10 μM), and 8.2 μL of sterile water. The qRT-PCR program began with an initial denaturation at 95°C for 30 s, followed by 40 cycles of 95°C for 10 s, 60°C for 30 s, and a melting curve at 95°C for 30 s, 60°C for 60 s, and 95°C for 15 s, three replicates were set for each sample. Both *actin beta* (*actb*) and *ubiquitin-conjugating enzyme* (*ubce*) genes were used as reference genes following the studies used in Nile tilapia by Deloffre et al. (2012), Yang et al. (2013), and Chen et al. (2020). The qRT-PCR specific primers were designed by Primer Premier 5 and are listed in Supplementary Table S2. A pretest of each primer pair is required to obtain a standard curve before the experiment. The standard curves of all genes were constructed using tilapia liver cDNA. The standard was diluted in five gradients as 1×, 5×, 25×, 125×, and 625×, and three replicates were set for each gradient, the system and reaction conditions are the same as above. Multiple primer pairs for each target gene were designed, and primers with *R* ^2^ between 0.99 and 1.00 was selected for the transcriptome validation experiments. The relative mRNA levels of target genes were calculated using the 2^−△△Ct^ method.

### Statistical Analysis

SPSS 24.0 software (Hansen, 2005) was used for Levene’s Test for Equality of Variances and Independent Samples *t*-test (Sedgwick, 2010) for growth traits (*n* = 45), biochemical parameters (*n* = 12), and qRT-PCR (*n* = 12) comparison between HG and LG in this study, results are presented as box plots providing sample distribution to clearly demonstrate the distribution of results and differences were considered significant at *p* < 0.01. To describe the clustering among sequenced samples and the differences in DEGs, M-versus-A plot (MA plot) were using ggpubr package, Principal component analysis (PCA) were using ggplot 2 package (Ginestet, 2011) and clusters samples based on their expression in all annotated genes according to Ren et al. (Ren et al., 2022), the above packages are based on R software (Version 4.1.0).

## Results

### Growth Comparison of HG and LG Groups

Growth comparison analysis using the 90 samples of extreme-growth individuals (45 from each the HG and LG groups) revealed significant differences in all growth traits (*p* < 0.01) (Figure 1B). In addition, significant differences were shown in WG, SGR, and ADG between the HG and LG fish (*p* < 0.01). However, there was no significant difference in CF (*p* > 0.05) (Figure 1C).

### Comparison of Biochemical Parameters Under Growth Differences

**FIGURE 2:**
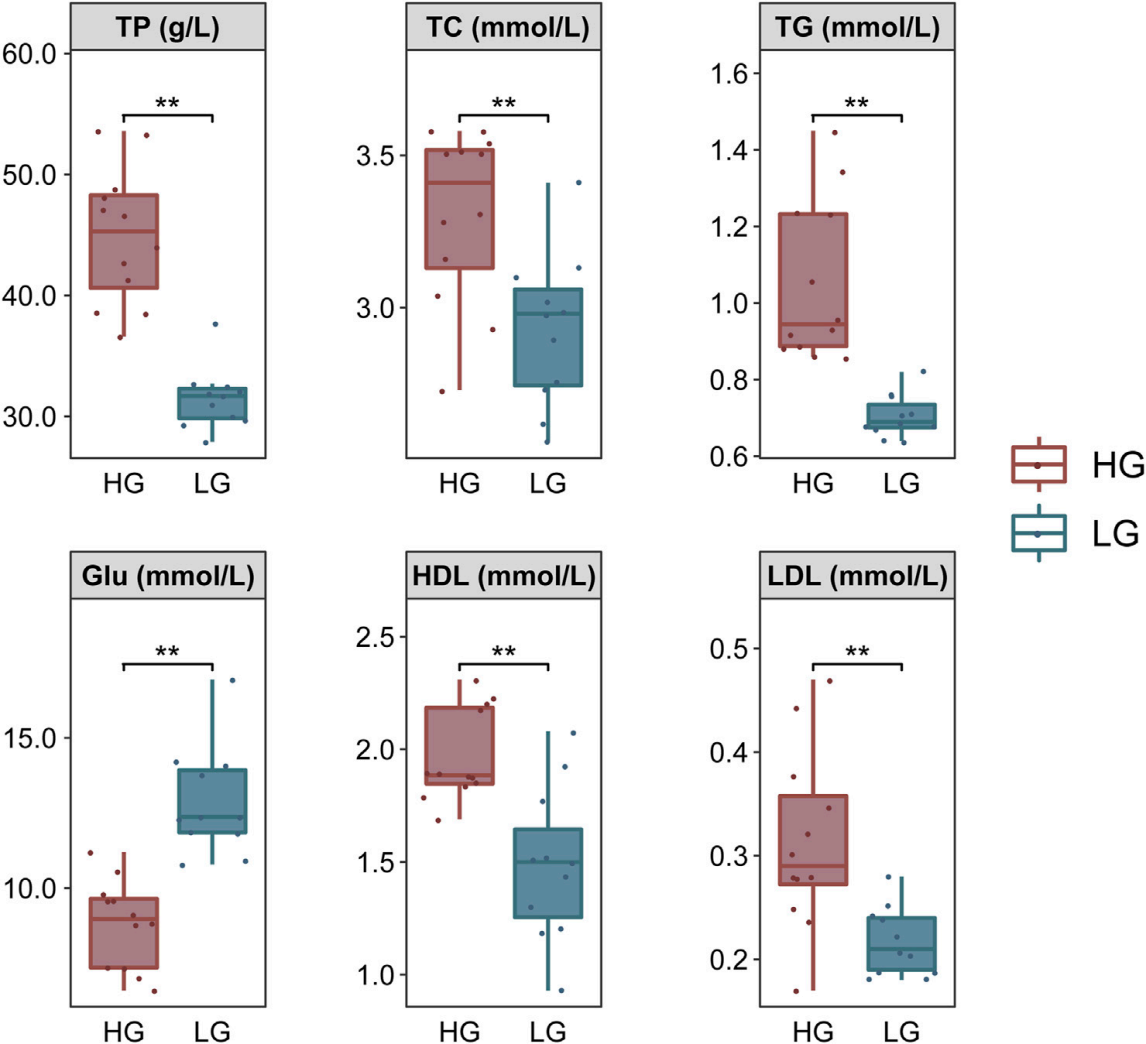
Comparison of biochemical parameters of Nile tilapia, grouped according to extreme growth differences (*n* = 12). Levene’s test was used to assess the equality of variances in the data, and the independent samples *t*-test showed a normal distribution (*p* > 0.05). **p < 0.01.

The TP, TC, TG, HDL, and LDL contents were significantly higher, but Glu was significantly lower, in HG individuals compared with LG individuals (*p* < 0.01) (Figure 2).

### Transcriptome Sequencing Quality Assessment and Identification of DEGs

**FIGURE 3:**
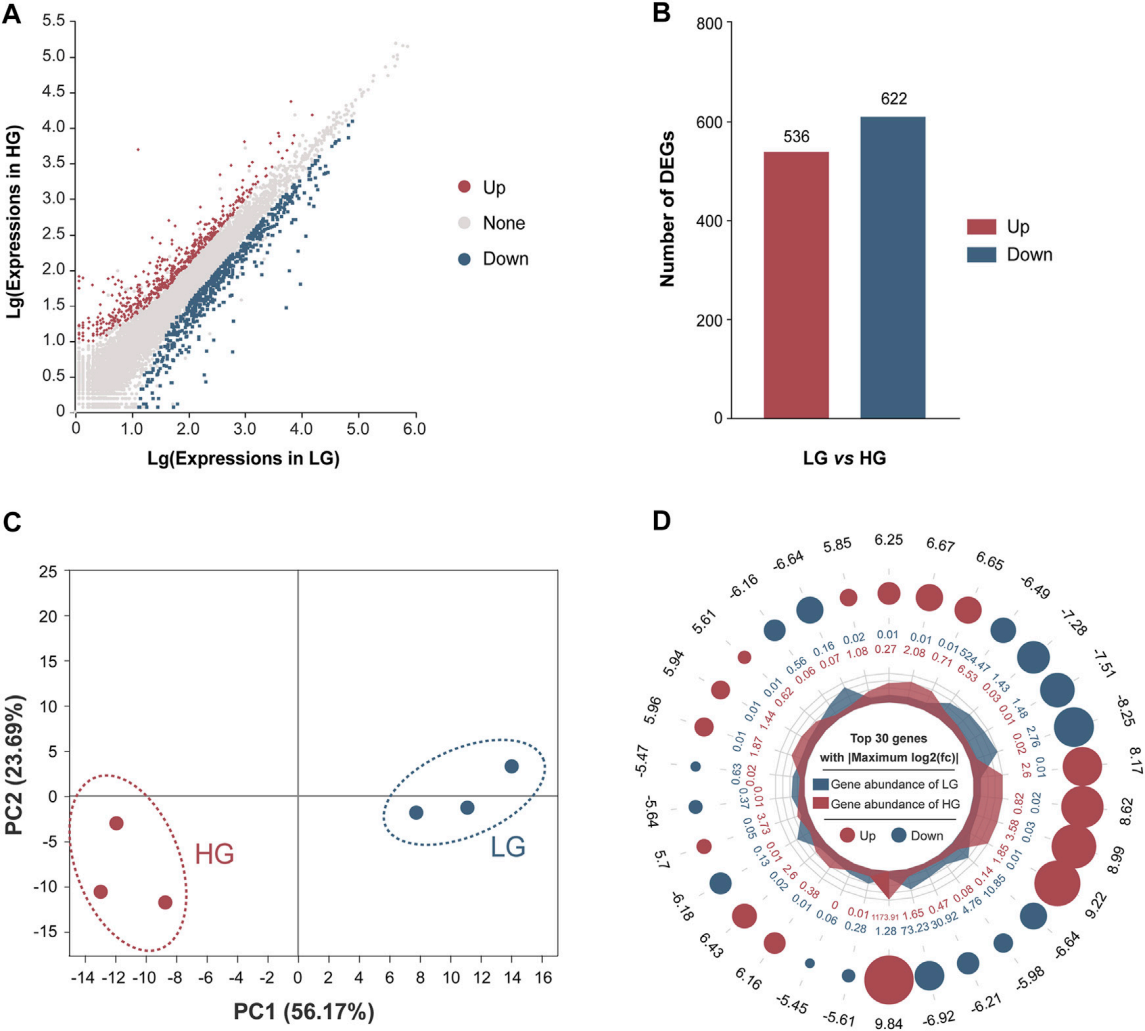
Comparison of differentially expressed genes (DEGs) between the higher growth (HG) and lower growth (LG) groups of Nile tilapia, with the LG group as the control. **(A)** M-versus-A plot (MA plot) of DEGs of the HG versus the LG group. **(B)** Numbers of DEGs between the LG and HG groups, with LG as the control group and HG as the comparison group, with log_2_ fold change (FC) of ≥2 or ≤0.5 (*p* < 0.01). **(C)** Principal components analysis of the HG and LG groups based on expression of all annotated genes. **(D)** Ploidy radar plot of the top-30 significantly different genes between the HG and LG groups. The numbers in the outermost circle are the differential multiples of genes; red and blue dots represent upregulation and downregulation, respectively; a larger circle diameter represents a larger absolute value of the multiples; the inner red and blue numbers represent the gene abundance within the HG and LG groups, respectively.

After filtering of raw data, the RNA sequence generated 44,991,790 to 51,295,092 clean reads in the liver of HG and LG. The Q20 and Q30 values for each sample were greater than 97.57 and 93.10%, respectively, and the average GC content was 48.98%. The alignment statistics results showed that the ratio of the mapped reads was approximately 91.92% when compared with the reference genome of Nile tilapia (Table 1). A total of 34,893 genes were annotated and used for DEG analysis, and a total of 1,158 DEGs were identified, including 536 upregulated genes and 622 downregulated genes (with LG as the control group and HG as the comparison group, FC ≥ 2 or ≤0.5; *p* < 0.01) (Figures 3A,B). Results of principal component analysis showed that the samples were clustered into two different groups according to high growth and low growth, which was consistent with the grouping of the samples (Figure 3C). Radar mapping showed that the multiplicity of the top-30 significantly upregulated or downregulated genes between the two groups ranged from 5.45 to 9.84 (Figure 3D). These results indicated that the data from these samples qualified for the DEG analysis.

**TABLE 1:** Statistics of total reads in RNA-Seq of higher growth group (HG) and lower growth group (LG) in the evaluation of superior growth performance in Nile tilapia (*Oreochromis niloticus*).

Sample	Raw Reads	Clean Reads	Q20 (%)	Q30 (%)	GC Content (%)
HG1	45,402,242	44,991,790	98.17	94.53	49.37
HG2	47,032,260	46,608,926	98.24	94.69	49.68
HG3	51,795,966	51,295,092	98.17	94.51	49.58
LG1	47,661,686	47,131,572	97.57	93.10	49.43
LG2	49,847,744	49,355,034	97.96	94.01	49.55
LG3	46,985,888	46,504,688	98.11	94.36	49.77

### Functional Analysis Using GO and KEGG Enrichment

**FIGURE 4:**
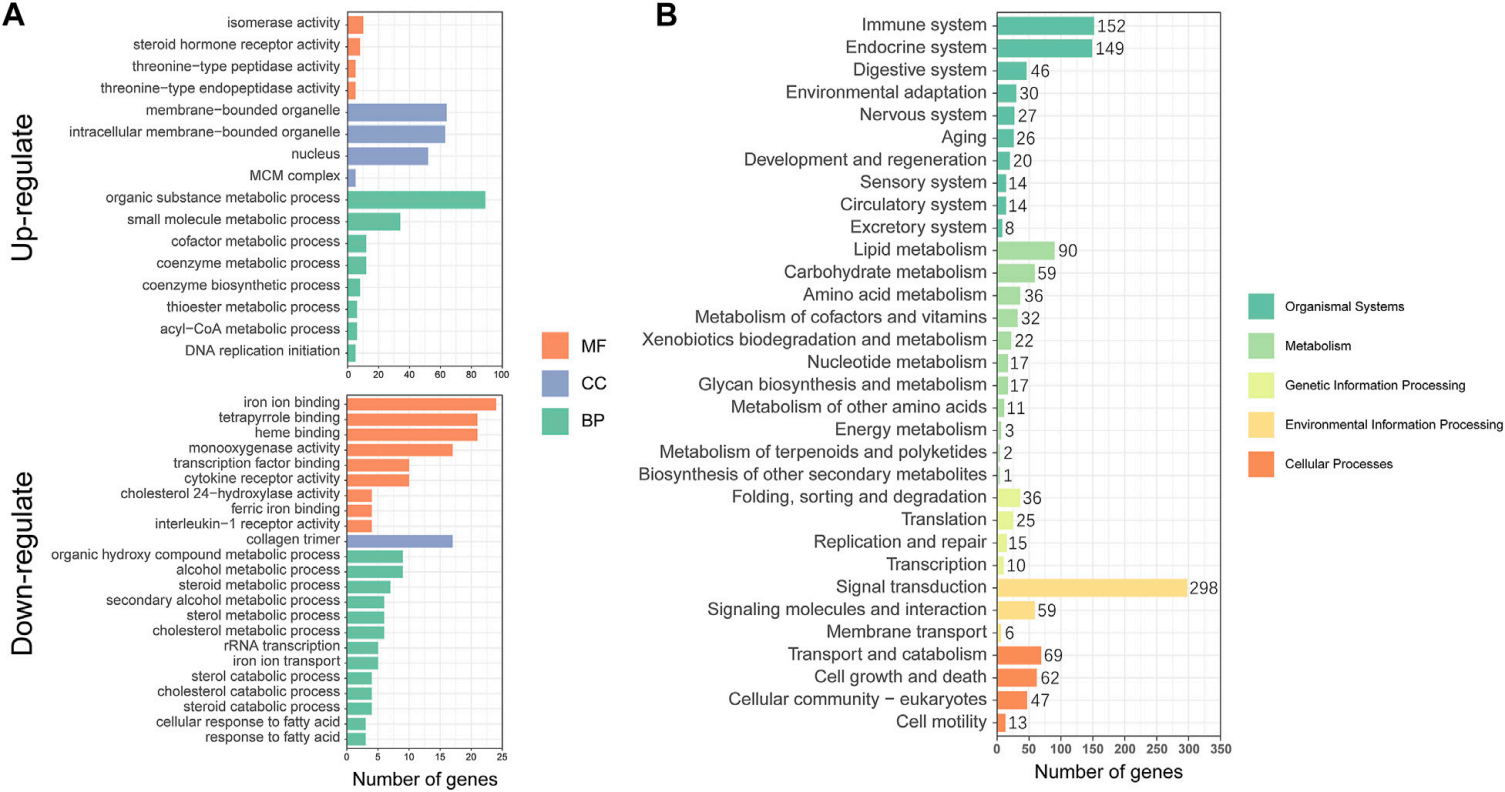
Functional annotation and enrichment analysis of differentially expressed genes (DEGs): **(A)** Gene Ontology (GO) functional enrichment analysis; **(B)** Kyoto Encyclopedia of Genes and Genomes (KEGG) functional annotation analysis.

To better understand the metabolic mechanisms in individuals that showed significant growth differences under the same culture environment and feeding regime, we performed GO enrichment analysis for the three main categories: molecular functions (MF), cellular components (CC), and biological processes (BP). The upregulated DEGs were mainly enriched in “organic substance metabolic process”, “small molecule metabolic process” of BP, “membrane-bounded organelle”, “intracellular membrane-bounded organelle”, “nucleus” of CC, and “isomerase activity”, and ‘steroid hormone receptor activity” of MF. The downregulated DEGs were mainly enriched in “iron ion binding”, “tetrapyrrole binding”, “heme binding” of MF, “collagen trimer” of CC and “alcohol metabolic process”, and “organic hydroxy compound metabolic process” of BP (Figure 4A). The KEGG annotation results showed that DEGs were concentrated in 32 pathways involving Cellular Processes, Environmental Information Processing, Genetic Information Processing, Metabolism, and Organismal Systems (Figure 4B).

**FIGURE 5:**
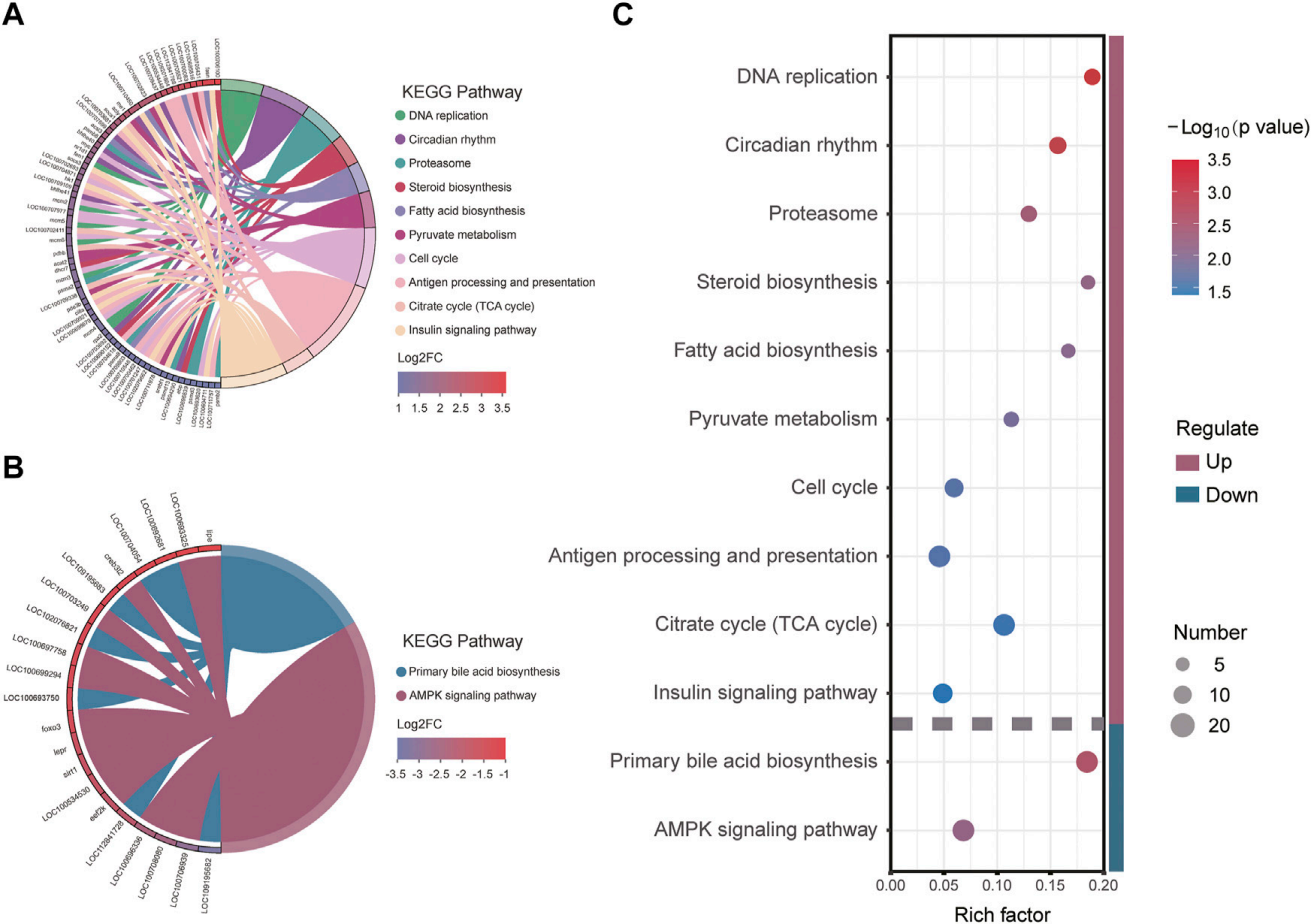
Kyoto Encyclopedia of Genes and Genomes (KEGG) pathway enrichment and its major enrichment genes: **(A)** upregulated differentially expressed genes (DEGs) significantly enriched; **(B)** downregulated DEGs significantly enriched; **(C)** significantly enriched KEGG pathways and the Rich factors.

To further elucidate the function of significant DEGs in signaling pathways, we annotated the DEGs significantly upregulated and downregulated in HG in the KEGG database to analyze the significantly enriched KEGG pathway. The results showed that three metabolism-related pathways were among the 10 pathways significantly enriched in upregulated DEGs (Figures 5A,C), namely Pyruvate metabolism, Citrate cycle (TCA cycle), Steroid biosynthesis, and Fatty acid biosynthesis. Cell cycle and DNA replication, two pathways associated with amplification of genetic information, were also defined as significantly enriched. Downregulated DEGs were significantly enriched in two pathways (Figures 5B,C), namely Primary bile acid biosynthesis and AMPK signaling (which is significantly associated with metabolic regulation). The above results suggest that the significantly enriched KEGG signaling pathway plays an important role in the metabolic system, and might be concentrated in three metabolic pathways: TCA cycle, Fatty acid biosynthesis and metabolism, and cholesterol biosynthesis and metabolism.

### Key Factors of the TCA Cycle Signaling Pathway in Superior Growth Performance Tilapia

**FIGURE 6:**
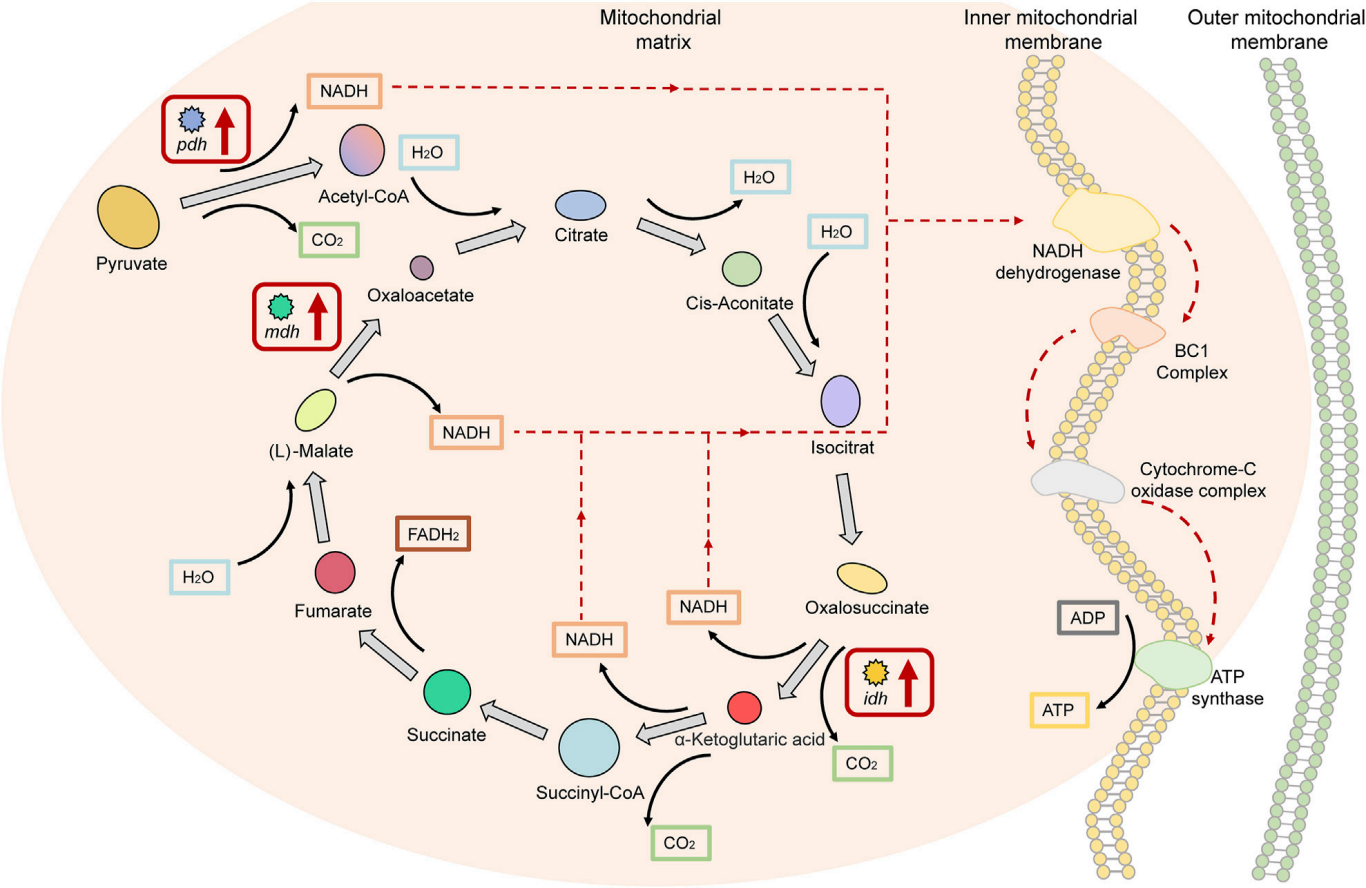
Key factors of the “tricarboxylic acid cycle (TCA cycle) signaling pathway” in superior growth performance Nile tilapia. Red boxes denote upregulated genes. Genes encoding key enzymes (*phd*, *idh*, and *mdh*) in pathways involved in energy metabolism (NADH-ATP) were upregulated.

Four genes in the upregulated DEGs of HG individuals were annotated to the TCA cycle signaling pathway, namely: *phda* and *pdhb*, encoding the pyruvate dehydrogenase E1 component, which promotes acetyl-CoA biosynthesis upstream of the TCA cycle; *idh*, encoding isocitrate dehydrogenase; and *mdh* encoding malate dehydrogenase, which is involved in the TCA cycle. The reactions involved in all three of these enzymes promote the production of nicotinamide adenine dinucleotide (NADH), which accounts for 75% of the total number of NADH synthesis pathways in the TCA cycle (Figure 6). These results indicate that genes encoding key enzymes in pathways involved in energy metabolism (NADH-ATP) were expressed as upregulated in Nile tilapia with superior growth performance.

### Key Factors of the Fatty Acid Biosynthesis and Metabolism Pathway in Superior Growth Performance Tilapia

**FIGURE 7:**
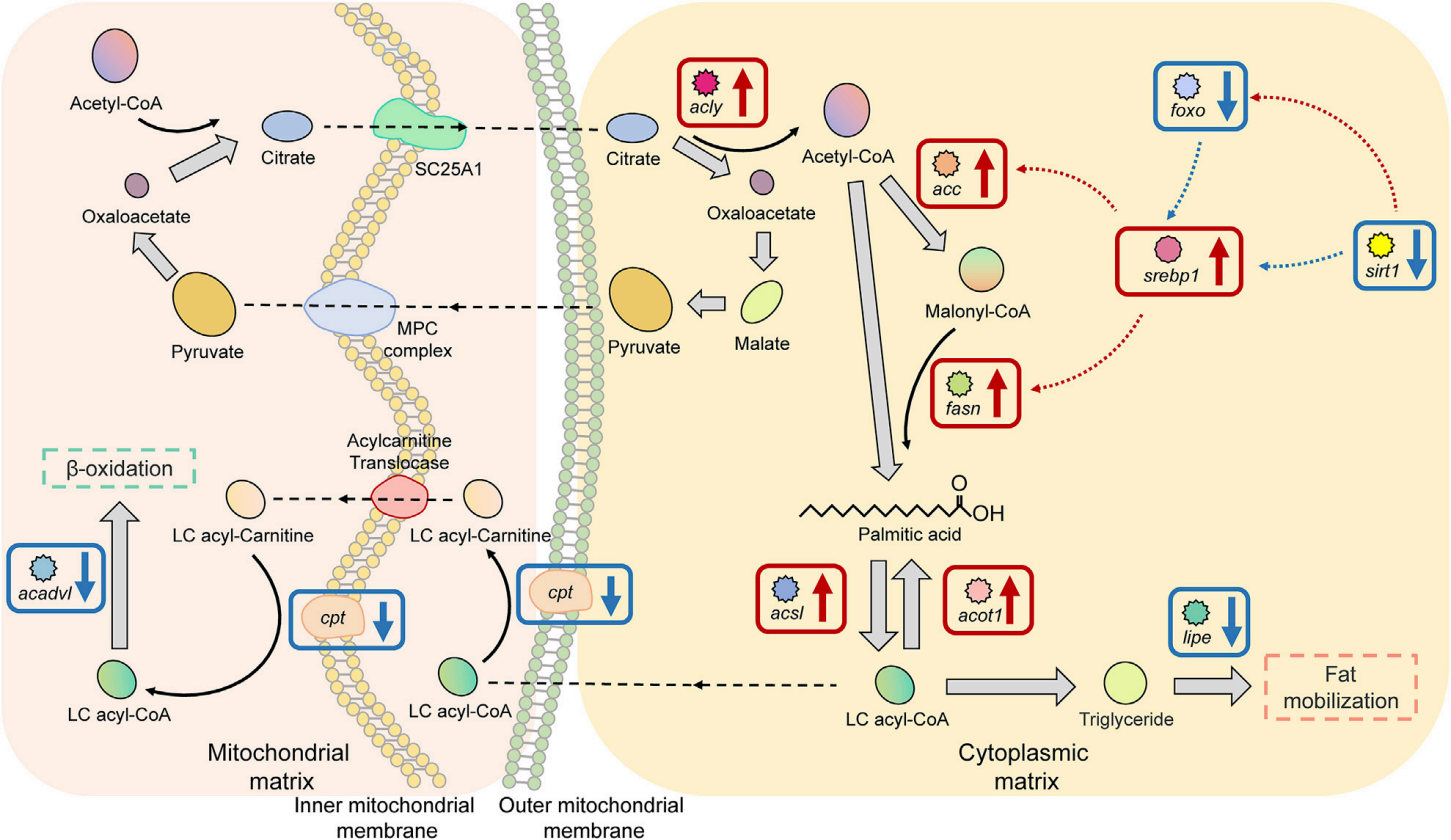
Key factors of the “fatty acid biosynthesis and metabolism pathway” in superior growth performance Nile tilapia. Red boxes mark upregulated genes, and blue boxes mark downregulated genes. Genes encoding key enzymes involved in the *ß*-oxidation pathway (*cpt*, *acadvl*) were downregulated, and genes encoding key enzymes involved in fatty acid biosynthesis and activation (*acly*, *acc*, *srebp1*, *fasn*, *acsl*, and *acot1*) were upregulated.

There were 11 significant DEGs annotated to the Fatty acid biosynthesis and metabolism pathway in HG individuals, of which the upregulated DEGs were ATP citrate lyase (*acly*), acetyl-CoA carboxylase (*acc*), fatty acid synthase (*fasn*), long-chain acyl-coenzyme A synthetase (*acsls*), acyl-coenzyme A thioesterase (*acot1*), and sterol-regulatory element-binding proteins 1 (*srebp1*); the proteins encoded by these genes are mainly used to promote fatty acid biosynthesis and to maintain the stability of free fatty acids. Downregulated DEGs were carnitine palmitoyl transferase 1a (*cpt1a*), hormone-sensitive lipase (*lipe*), NAD^+^-dependent protein deacetylase sirtuin 1 (*sirt1*), very long-chain acyl-CoA dehydrogenase (*acadvl*), and forkhead box O protein (*foxos*). The functions of the proteins encoded by these genes are different, as considered in the Discussion (Figure 7).

### Key Factors of the Cholesterol Biosynthesis and Metabolism Pathway in Superior Growth Performance Tilapia

**FIGURE 8:**
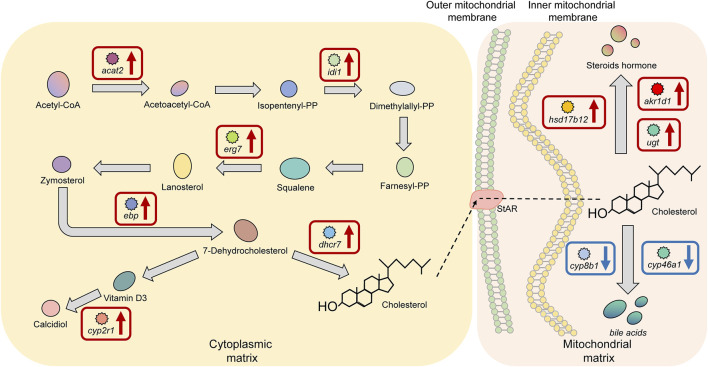
Key factors of the “cholesterol biosynthesis and metabolism pathway” in superior growth performance Nile tilapia. Red boxes mark upregulated genes, and blue boxes mark downregulated genes. Genes encoding key enzymes involved in synthesis of bile acids from cholesterol (*cyp8b1*, *cyp46a1*) were downregulated, and genes encoding key enzymes involved in synthesis of steroid hormones from cholesterol (*hsd17b12*, *akr1d1*, *ugt*) were upregulated.

There were 10 significant DEGs annotated to the cholesterol biosynthesis and metabolism pathway in HG individuals, including the upregulated genes acetyl-CoA acetyltransferase 2 (*acat2*), isopentenyl-diphosphate delta isomerase 1 (*idi1*), lanosterol synthase (*erg7*), cholesterol delta-isomerase (*ebp*), and 7-dehydrocholesterol reductase (*dhcr7*), which encode proteins or enzymes that are primarily used to promote cholesterol biosynthesis. The proteins or enzymes encoded by the downregulated genes, including hydroxysteroid dehydrogenase type 12 (*hsd17b12*), steroid 5β-reductases (*akr1d1*), and UDP-glucuronosyltransferase (*ugt*) are mainly used to promote steroid hormone biosynthesis, and the enzymes encoded by sterol 12alpha-hydroxylase (*cyp8b1*) and cholesterol-24S-hydroxylase (*cyp46a1*) are mainly used to promote the biosynthesis of bile acids (Figure 8).

### Correlation Analysis of Growth Performance, DEGs, and Biochemical Parameters

**FIGURE 9:**
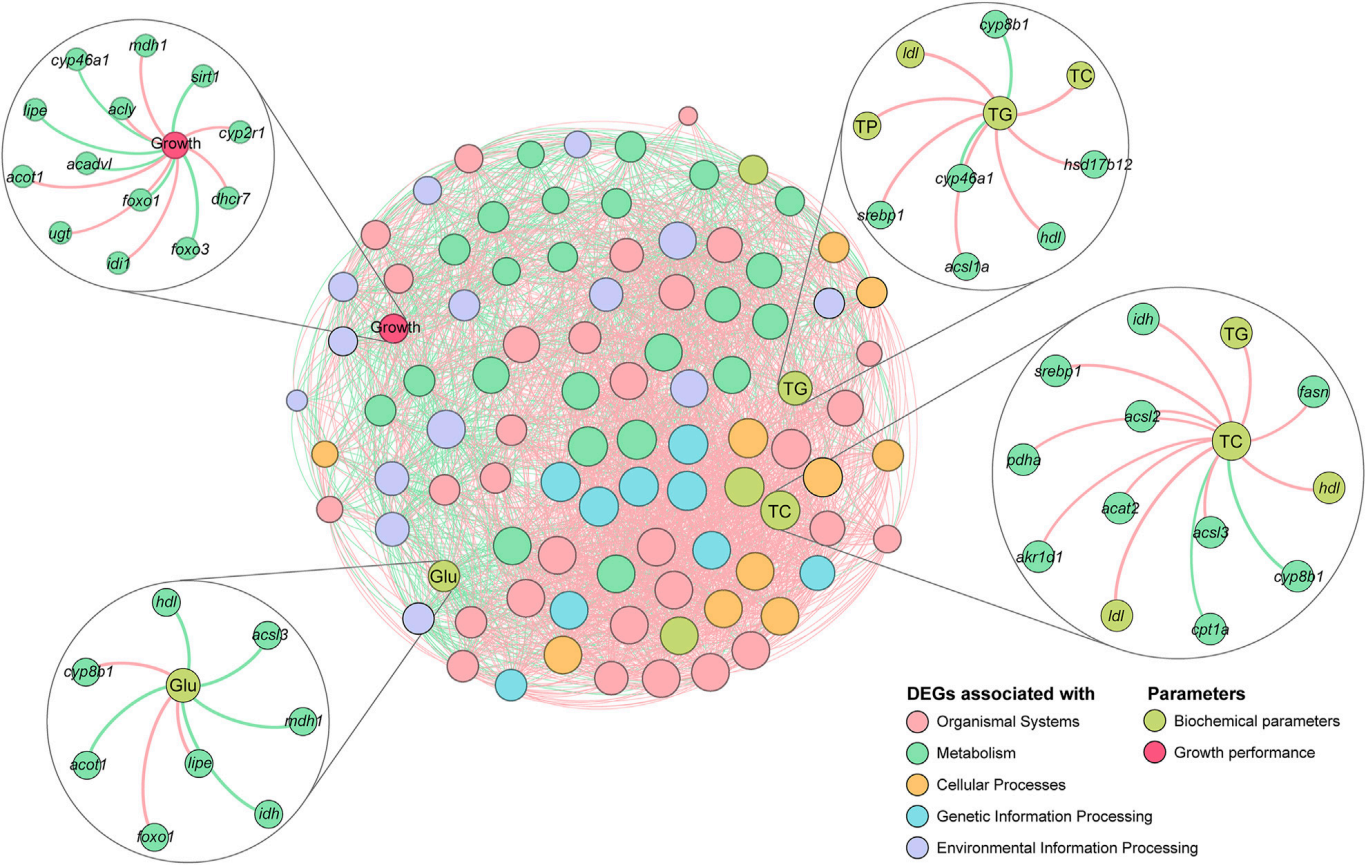
Correlation network analysis of tilapia growth performance, differentially expressed genes (DEGs), and biochemical parameters. The colors of the nodes indicate data types, and the sizes of the nodes indicate the degree in the network. Positive and negative correlations are represented by red and green lines, respectively.

Correlations between growth performance, DEGs, and biochemical parameters were further investigated. *mdh1* in the TCA cycle signaling pathway, *acly* and *acot1* in the Fatty acid biosynthesis and metabolism pathway, and *idi1*, *ugt*, *dhcr7*, *cyp2r1*, and *cyp46a1* in the cholesterol biosynthesis and metabolism pathway showed significant positive correlations with growth performance, and the correlations were consistent with the results at the transcriptional level, indicating that the expression levels of these genes increased with growth performance of the tilapia. In contrast, *sirt1*, *foxo1*, *foxo3*, *lipe*, *acadvl*, and *cyp46a1* were negatively correlated with growth performance, indicating that the expression levels of these genes decreased with an increase in the growth performance of the tilapia. In the correlation analysis with Glu, the expression of *idh* and *mdh1* showed significant negative correlation with the concentration of Glu, indicating that as the expression of those genes increases, the concentration of Glu decreases, which is consistent with the function of these genes (consumption of Glu in energy metabolism). In the correlation analysis of TC and TG, the correlation trends of the genes were consistent with the transcriptional results, indicating that the above genes significantly influenced lipid metabolism and sterol metabolism in the tilapia. Notably, *srebp1* and *acsls* showed significant positive correlations with both TC and TG, while *cyp8b1* showed significant negative correlations with both TC and TG, which may indicate that these genes play an important role in the metabolic process (Figure 9).

### Quantitative Real-Time PCR Analysis

**FIGURE 10:**
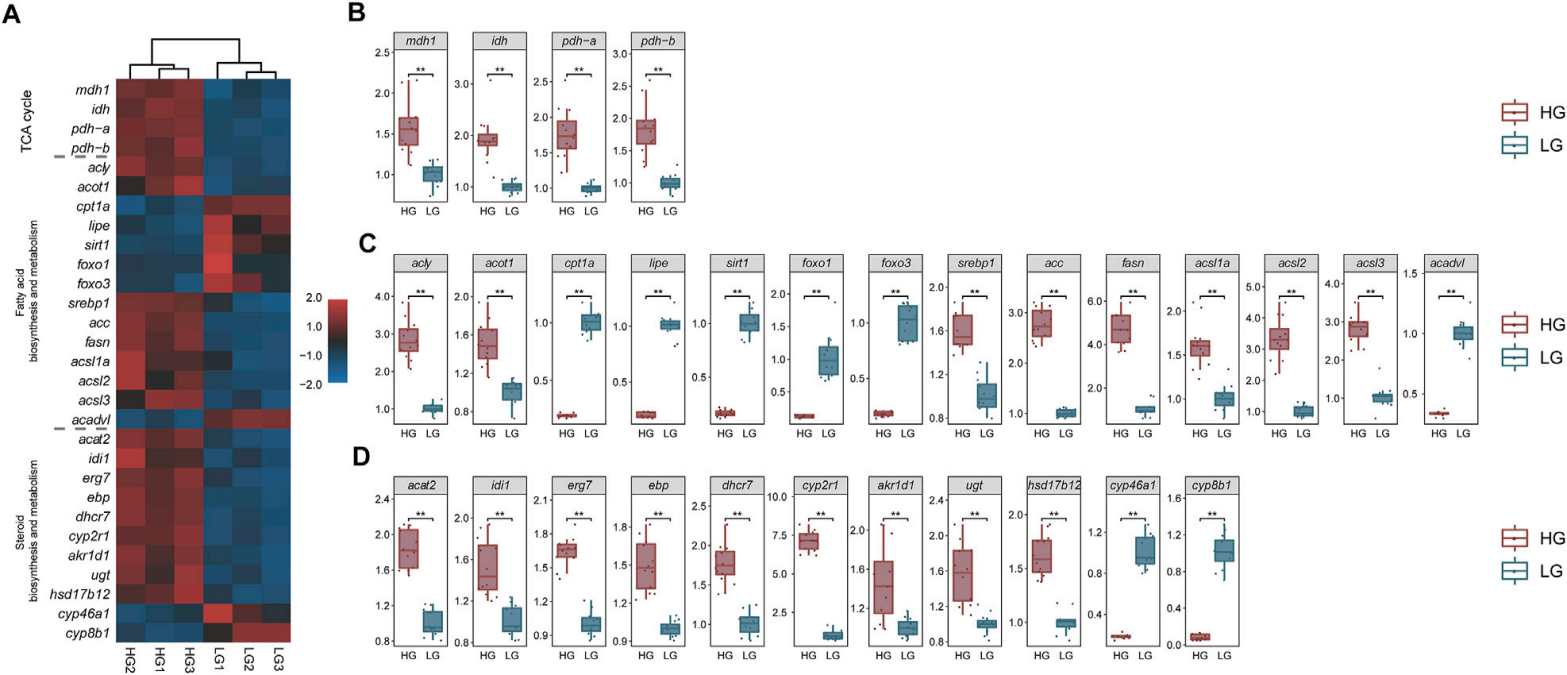
Transcriptome transcripts per million (TPM) values (*n* = 3) and qRT-PCR results of key genes (*n* = 12). **(A)** Heat map of transcriptome TPM values of key genes; mRNA levels of key genes in **(B)** the “TCA cycle pathway”, **(C)** the “fatty acid biosynthesis and metabolism pathway”, and **(D)** the “cholesterol biosynthesis and metabolism pathway”. **p < 0.01.

The qRT-PCR results showed that the relative expression of each key gene was in line with the trend in the TPM values. Key genes in all three signaling pathways (TCA cycle, Fatty acid biosynthesis and metabolism, and cholesterol biosynthesis and metabolism) showed significant differences between HG and LG tilapia (*p* < 0.01) (Figure 10).

## Discussion

Recent research on tilapia growth performance has focused on feed and additives (Zhou et al., 2010; Genschick et al., 2021; Yu et al., 2021), managing the environment (Charo-Karisa et al., 2006; Turra et al., 2016), culturing models (Zhang et al., 2018; Ani et al., 2021), and growth-related molecular markers (Khatab et al., 2014; Chen et al., 2020). Among these, the more relevant study for growth performance enhancement was the screening of growth-related QTLs in tilapia (Yoshida and Yáñez, 2021), but the 29 metabolism-related candidate genes obtained in the present study were not among them. These different outcomes may be attributable to the exploration levels: identification of the QTLs was on the genomic level in the work of Yoshida and Yáñez (2021), while in our study it was based on the transcriptome level. In addition, the candidate genes obtained from growth-related QTLs were obtained through correlation analysis, whereas the transcriptome results were obtained based on analysis of significant differences. No studies to date have reported on tilapia with the same genetic background but with significant growth differences under the same culture environment and feeding method. In wild-type aquatic animals, gene expression between different growth performance groups has been reported for vannamei shrimp (*Litopenaeus vannamei*) (Santos et al., 2021) and California red abalone (*Haliotis rufescens*) (Valenzuela-Miranda et al., 2015). To some extent, these studies have revealed the relationship between growth performance differences and genes in aquatic animals; however, the available research is insufficient to elucidate the mechanisms of transcriptome-level regulation of growth differences in wild-type bony fishes, especially cichliforms. In a study of non-wild type coho salmon (*Oncorhynchus keta*), McClelland et al. (2020) found that comparative transcriptome results between GH transgenic (T) and non-transgenic (NT) individuals with different growth performance (T_large_, T_small_, NT_large_, and NT_small_) indicated that there are widespread regulatory influences acting to influence body size and gene expression traits, in addition to the effects of GH transgenesis. Those authors also showed that multiple regulatory loci affecting gene expression were shared between fast-growing and slow-growing fish within T or NT groups, but no such regulatory loci were found to be shared between those two groups (McClelland et al., 2020). This demonstrates the complexity and important research potential of gene expression differences among individuals with different growth performance.

Without a reference genome, DEGs can only be compared by splicing into unigenes or by annotation to databases, such as Non-Redundant Protein Sequence Database (NR), GO, and KEGG, which drastically reduces the number of actual DEGs (Zhang et al., 2019). This implicates the importance of a species’ reference genome for comparative transcriptomic analyses. We annotated RNA-seq data into the tilapia reference genome and identified 1,158 DEGs; this availability increases the significance and representativeness of our results. The influence of enzymes (i.e., changes in the activity of the enzyme itself) on the metabolic pathway depends on the changes in the activity of the entire metabolic pathway (Rose, 1968; Bell, 2012). For example, an enzyme with a significant change in activity cannot affect the entire metabolic pathway if the change does not affect other processes in the pathway; conversely, if most enzymes in a metabolic pathway show significant changes in activity, then the entire metabolic pathway is affected (either stimulated or inhibited). Our results show that the significantly upregulated and downregulated DEGs were enriched within the three metabolic pathways, and the expression levels of a proportion of genes were significantly changed within each pathway (Figures 6–8). This result is consistent with the above view and further strengthens our speculation that these metabolism-related enzymes or proteins play a key role in body weight gain in Nile tilapia while constituting potential candidate genes at the transcriptional level. Therefore, in the following sections we discuss the three key metabolic pathways in detail, namely the TCA cycle, Fatty acid biosynthesis and metabolism, and cholesterol biosynthesis and metabolism.

### Mechanisms of the TCA Cycle Signaling Pathway in Tilapia With Superior Growth Performance

The TCA cycle is the main process for the metabolism of sugars, lipids, and amino acids in the mitochondria and is the metabolic hub in mast eukaryotes (Ryan and O’Neill, 2020). The TCA cycle starts with the oxidative decarboxylation of pyruvate; the resulting acetyl-CoA is regenerated to oxaloacetate and CO_2_ after four dehydrogenation reactions; and the whole process takes place within a cycle of eight main reactions (Krebs and Johnson, 1980). NADH and flavin adenine dinucleotide (FADH), which are produced during the TCA cycle, put it at the center of energy metabolism. NADH and FADH in the inner mitochondrial membrane transfer electrons to O_2_ *via* an electron transport chain containing NADH dehydrogenase, BC1 complex, and cytochrome-C oxidase complex, and transport protons to the mitochondrial intermembrane space to generate transmembrane proton gradients and electrical potential for the synthesis of ADP to ATP *via* ATP synthase (Saraste, 1999). The ATP produced by the TCA cycle is mainly from NADH, which accounts for approximately 75% of the total (Pelley, 2007). Studies have reported that increasing the concentration of NADH significantly increased the ATP synthesis capacity of cardiomyocytes (Pelzmann et al., 2003); this suggests that the cell’s ability to synthesize ATP is positively correlated with its ability to produce NADH in the TCA cycle. The number of molecules of NADH that can be produced in one TCA cycle is 3; hence, the cell’s ability to produce ATP is related to the rate, or flux, of the TCA cycle.

Our results showed that the genes encoding Pdh, Idh, and Mdh were significantly upregulated in Nile tilapia with superior growth performance. Pdh has long been considered a key factor in the entry of pyruvate into the TCA cycle, and it plays an important catalytic role in the synthesis of acetyl-CoA from pyruvate, which is the only way for mammals to synthesize pyruvate into acetyl-CoA (Kim et al., 2006; Tennant et al., 2010). As the first enzyme involved in the TCA cycle, its activity directly affects the rate of the cycle (Whitehouse et al., 1974). Idh is one of the key enzymes in the TCA cycle, wherein isocitrate is converted to oxalosuccinate and rapidly decarboxylated to *a*-ketoglutaric acid; because this reaction is irreversible, Idh is the key rate-limiting enzyme in the TCA cycle (Xu et al., 2004; Leonardi et al., 2012). In the final step of the cycle, the oxaloacetate initially consumed is re-synthesized by (L)-malate and catalyzed by Mdh, which is a key step in making the TCA cycle a closed loop (van der Rest et al., 2000). This suggests that increased Pdh, Idh, and Mdh activity may be the main way by which cells regulate the increased flux of the TCA cycle. Interestingly, we found that the reactions in which Pdh, Idh, and Mdh participate in the TCA cycle all catalyze the production of NADH, the most critical substrate promoting the conversion of ADP to ATP. Our method of providing adequate feed for each experimental fish excluded a decrease in Glu caused by inadequate food intake. As noted above, the concentration of Glu in the HG group of tilapia was significantly lower than in the LG group, combined with the significant upregulation of *pdh*, *idh*, and *mdh* in the superior growth performance tilapia. We suggest that excessive Glu consumption in superior growth performance tilapia may be related to Pdh, Idh, and Mdh and the TCA cycle in which they are located. The specific regulatory processes of which need to be further investigation.

### Mechanisms of the Fatty Acid Biosynthesis and Metabolism Pathway in Tilapia With Superior Growth Performance

Fatty acids, the main components of many key substances such as fats, phospholipids, and glycolipids, are the main source of energy for most organisms (Dole and Meinertz, 1960), and their biosynthesis is facilitated when the organism needs to obtain energy or to carry out energy storage (Gibson, 1963). Fatty acids are synthesized from acetyl-CoA in a process that occurs in the cytoplasm and is catalyzed by several enzymes; however, acetyl-CoA is produced in the mitochondria and cannot cross the mitochondrial membrane directly, so it is first bound to oxaloacetate to produce citrate, which can enter the cytoplasm *via* the citrate transporter protein (Slc25a1) and is subsequently reconverted to oxaloacetate and acetyl-CoA catalyzed by Acly (Burke and Huff, 2017). In the initial step of fatty acid biosynthesis, acetyl-CoA is synthesized into malonyl-CoA catalyzed by Acc, which is the main form of acetyl-CoA participating in fatty acid synthesis, and therefore this process is an important rate-limiting step (Park et al., 2002; Saggerson, 2008). In fatty acid biosynthesis in animal cells, acetyl-CoA is used as the starting fragment and may be extended seven times by the addition of two carbon atoms (malonyl-CoA) to the carboxyl terminus to produce palmityl ACP, through a process catalyzed by Fasn (Chavin et al., 2004). Palmityl ACP is catalyzed by palmityl ACP thioesterase to release palmitic acid, which completes the entire fatty acid biosynthesis process (Jones et al., 1995). In our results, the genes encoding Acly, Acc, and Fasn, which are key enzymes involved in fatty acid biosynthesis, were significantly upregulated in tilapia with superior growth performance. This suggests a higher capacity for fatty acid biosynthesis in those fish. In addition, we identified changes in some of the transcription factors that can be involved in fatty acid biosynthesis in superior growth performance tilapia. Among them, *srebp-1* appeared significantly upregulated, whereas *foxos* and *sirt1* were significantly downregulated. The current research suggests that Srebp-1 promotes transcription of *acc* and *fasn* (Wang et al., 1994). Although the role of Foxo in the regulation of lipid metabolism is not fully understood, it has been suggested that it can inhibit the expression of *srebp-1* (Deng et al., 2012). Sirt1 is an important regulator at the center of several lipid metabolism networks (Picard et al., 2004); numerous studies have shown that it can regulate the activity of various transcription factors or enzymes that play an important role in fatty acid synthesis and *ß*-oxidation, such as Srebps and Foxos, by deacetylating target proteins (Wang et al., 2017). There was a significant reduction in cellular beta-oxidation capacity and concomitant hyperlipidemia after specific deletion of Sirt1 in mouse (*Mus musculus*) (Walker et al., 2010). Thus, *via* the upregulation of *srebp-1* and the downregulation of *foxos*, *sirt1* could produce the same final effect (i.e., both promote fatty acid biosynthesis, which provides more support for our hypothesis).

Once fatty acid has been synthesized it must be activated (bind to CoA to synthesize acyl-CoA) to enter the metabolic pathway, which is the first step in the participation of fatty acids in biological reactions (Færgeman and Knudsen, 1997), and this process is catalyzed by the acyl-CoA synthetase family. In particular, Acsl specifically catalyzes the synthesis of long-chain acyl-CoA (LC acyl-CoA) from fatty acids between C12 and C22 and further participates in catabolism or anabolism (Coleman et al., 2000). The main pathway of LC acyl-CoA catabolism is *ß*-oxidation, a process that occurs within the mitochondria. To cross the mitochondrial inner membrane, LC acyl-CoA needs to bind carnitine to synthesize LC acyl-carnitine catalyzed by Cpt, and when it passes through the mitochondrial inner membrane it will release carnitine to convert to LC acyl-CoA catalyzed by Cpt (Townsend et al., 2013). Upon entry into the mitochondria, LC acyl-CoA progressively releases acetyl-CoA, the precursor substance for ATP synthesis in the TCA cycle pathway, and this process is catalyzed by Acadvl (Zhang et al., 2007). The activity of Acot1 is activated when the intracellular ATP concentration is gradually increased. Acot1 reduces the concentration of substrates involved in ATP synthesis by hydrolyzing acyl-CoA in the cytoplasm to free fatty acids and CoA to avoid the waste of energy caused by excessive ATP synthesis (Zhang et al., 2012).

Changes in the activity of the above enzymes constitute the main pathway for bioregulation of *ß*-oxidation. In this study, we found that *acsls* were significantly upregulated but *cpt1a* and *acadvl* were significantly downregulated in superior growth performance tilapia, suggesting that these tilapia have a higher capacity for fatty acid activation, but the *ß*-oxidation process is inhibited. Meanwhile, *acot1* was significantly upregulated, and, since the activity of Acot1 is only activated at high energy levels (Franklin et al., 2017), this implies that superior growth performance tilapia maintain high energy levels despite the inhibition of the *ß*-oxidation pathway, which could suggest that energy acquisition in superior growth performance tilapia is not derived from the catabolism of fatty acids (β-oxidation). In addition, we found that *lipe* was significantly downregulated. Lipe is thought to be a key enzyme in mobilizing TG deposited in adipose tissue, which hydrolyses TG to free fatty acids, and is also the rate-limiting enzyme in the hydrolysis of diglycerides (Haemmerle et al., 2002; Zimmermann et al., 2004). This finding, combined with the significantly higher TG concentrations in the HG tilapia compared with in the LG fish suggests that the activated fatty acids in superior growth performance tilapia are used more for TG synthesis than for *ß*-oxidation. Moreover, the inhibition of the catabolic pathway of TG implies that its storage capacity is improved.

An integrated description of the above results suggests that superior growth performance tilapia may have a high capacity for fatty acid synthesis and activation, but that the *ß*-oxidation pathway is inhibited without a concomitant reduction in cellular energy levels, thus the fish may then be able to synthesize and store TG under the influence of higher energy levels. In short, we suggest that Nile tilapia with superior growth performance may have lower activity in the fatty acid catabolic pathway but higher activity in the anabolic pathway. Consequently, the large amount of synthesized fatty acids may be used more for TG synthesis after activation, to store excess energy.

### Mechanisms of the Cholesterol Biosynthesis and Metabolism Pathway in Tilapia With Superior Growth Performance

Cholesterol is the most abundant sterol compound in nature and is one of the most important lipids in the human body (Brown and Goldstein, 1997). The biosynthesis of cholesterol is complex, with nearly 30 enzymatic steps (Stocco and Clark, 1994), which can be divided into three main stages: the synthesis of isopentenyl pyrophosphate (IPP) from acetyl CoA; the conversion of IPP to squalene; and the conversion of squalene to cholesterol. In our study, superior growth performance tilapia showed upregulation of genes encoding key enzymes within all these stages. Combined with the results we reported here, where the concentration of TC was significantly higher in the HG group than in the LG tilapia, this may suggest a higher capacity for cholesterol biosynthesis in superior growth performance tilapia.

Unlike fatty acids, cholesterol cannot be degraded to CO_2_ and H_2_O but is oxidized to become important bioactive substances, such as bile acids, steroid hormones, and vitamin D3 (Goldstein and Brown, 1990). Bile acids are the main product of cholesterol metabolism in the liver. After being synthesized, they are concentrated and stored in the gallbladder and participate in the digestion and absorption of lipids and fat-soluble vitamins after food intake (Li and Chiang, 2014; Di Ciaula et al., 2017). Bile acids play an important regulatory role in TG and lipid metabolism; in mouse, increasing the number of circulating bile acids improved high-fat diet-induced obesity (Harach et al., 2012; Kohli et al., 2013). Steroid hormones are another important endogenous substance synthesized from cholesterol and can be classified as corticosteroids and sex hormones. Adrenocorticotropic hormones are synthesized from cholesterol in the zona glomerulosa of the adrenal cortex and can promote gluconeogenesis and protein metabolism and increase the synthesis of liver glycogen and muscle glycogen (Campbell, 2011). Sex hormones are synthesized in the zona reticularis of the adrenal cortex and transformed into dihydrotestosterone or estradiol in the testes or ovaries, where they play a physiological role in promoting protein synthesis, especially in the muscle and reproductive organs, as well as bone growth (Gorski and Gannon, 1976). Numerous studies have shown that sex hormones in humans promote the secretion of growth hormone (GH) and mediate the GH-IGF1 signaling pathway to influence the development and growth of bones and muscles in adolescents (Tanner et al., 1976; Holmes and Shalet, 1996; Mauras et al., 1996). In studies on tilapia, it was found that sex hormones may mediate growth hormone-releasing hormone to affect regulation of tilapia growth (Melamed et al., 1995), suggesting the sex hormones that regulate growth in tilapia are similar to those humans. In our study, *cyp8b1* and *cyp46a1* were significantly downregulated, and *hsd17b12*, *akr1d1*, and *ugt* were significantly upregulated in the livers of superior growth performance tilapia. Among these, Cyp8b1 and Cyp46a1 are key regulators of cholesterol synthesis into bile acids (Savolainen et al., 2004; Yang et al., 2004), while Hsd17b12, Akr1d1, and Ugt play key roles in several steps of cholesterol synthesis into steroid hormone precursors (Lima et al., 2013; Chen et al., 2019). Together these results may suggest that superior growth performance Nile tilapia have a lower capacity for cholesterol synthesis for conversion into bile acids and a higher capacity for synthesis towards steroid hormone precursors.

### Other Key Factors That May Be Involved in the Regulation of Tilapia Metabolism

The growth hormone-insulin like growth factor1 (GH-IGF1) is a key factor affecting animal growth. Previous studies showed that the growth-promoting effect of Igf1 may depend on its feedback regulation of starvation (Rousseau and Dufour, 2007), such as in rainbow trout (*Oncorhynchus mykiss*) (Niu et al., 1993), channel catfish (*Ictalurus punctatus*) (Small and Peterson, 2005), coho salmon (Moriyama et al., 1994), and tilapia (Uchida et al., 2003). However, in this study, all individuals were fed to apparent satiation (see 2.1 for details), therefore the regulatory role of Igf1 on tilapia growth *via* starvation feedback (to promote food intake) may have been reduced; this might explain why Igf1, and some other appetite-related factors such as *ghrelin* and *prepro-orexin* (Unniappan et al., 2004; Novak et al., 2005), were not screened in this study.

It has been reported that Gh can indirectly participate in metabolic processes in animals by regulating downstream factors, including stimulation of lipid mobilization, and induction of protein synthesis (Sheridan, 1994; Björnsson, 1997). In addition, some key genes located in the hypothalamus, such as pituitary adenylate cyclase activating polypeptide (*pacap*), corticotropin-releasing hormone (*chr*), and thyrotropin-releasing hormone (*trh*), also might be indirectly involved in the metabolic regulation of animals as upstream factors (Gershengorn, 1982; Rousseau and Dufour, 1999; Montero et al., 2000). In our study, many genes related to lipid regulation (*acc*, *srebp1*, *sirt1*, *fasn*, *acsl*, *acot1*, *lipe*) and protein synthesis (*hsd17b12*, *akr1d1*, *ugt*, *cyp8b1*, *cyp46a1*) were identified, and the variability of these factors may be related to regulation of the upstream genes mentioned above. The metabolism-related genes obtained in this study were mainly in the liver; accordingly, these upstream key factors were not screened, yet their role in the regulation of metabolism cannot be ignored, and the regulatory mechanisms of metabolism-related genes between upstream and downstream need to be further investigated.

## Conclusion

This study used biochemical and transcriptome analyses to compare individuals of Nile tilapia to reveal significant growth differences under the same culture environment and feeding regime. The results showed that the contents of TP, TC, TG, HDL, and LDL were significantly increased, while Glu was significantly reduced in the superior growth performance individuals. Furthermore, 29 metabolism-related candidate genes were obtained and showed significant differential expression in the superior growth performance tilapia. The possible relationships between these genes were described, and three potential metabolic pathways were mapped. To our knowledge, this is the first study to describe the potential metabolic characterization of superior growth performance Nile tilapia obtained from full-sibling families reared in the same culture environment; our findings provide more information and ideas for improved growth performance of farmed tilapia.

## Data Availability

The original contributions presented in the study are publicly available. This data can be found here: PRJNA787719.
